# It Takes a Village to Raise a Child: Understanding and Expanding the Concept of the “Village”

**DOI:** 10.3389/fpubh.2022.756066

**Published:** 2022-03-11

**Authors:** Andrea Reupert, Shulamith Lala Straussner, Bente Weimand, Darryl Maybery

**Affiliations:** ^1^Faculty of Education, Monash University, Clayton, VIC, Australia; ^2^Silver School of Social Work, New York University, New York City, NY, United States; ^3^Faculty of Health and Social Sciences, University of South-Eastern Norway, Drammen, Norway; ^4^Division of Mental Health Services, Akershus University Hospital, Lørenskog, Norway; ^5^School of Rural Health, Monash University, Warragul, VIC, Australia

**Keywords:** perspective, community, children, parent, adversity, parents, caregivers

## Abstract

This perspective article defines and discusses the concept of the “village” when working with families who are experiencing multiple adversities. The article starts with a discussion on what is meant generally by a village approach, followed by a historical overview of how families living in adversity have been defined and positioned. The need to move past a siloed, professional centric approach when working with families is then presented. Using a model of social connections, based on Bronfenbrenner's ecological theory, we then identify who the “villagers” might be. Some potential principles for how the village might work with families living with adversity are presented, along with two case studies, to demonstrate how these principles might be enacted. This perspective article provides an overview and discussion of “the village” concept, rather than present a definitive set of guidelines or recommendations.

## Introduction

Globally, many families face multiple adversities. These advertises may include mental illness, substance use and addiction problems, physical illness, domestic and community violence, poverty, insecure housing and war. Moreover, many of these problems are accumulative, with one problem, for example, parental mental illness, cascading into other problems, such as relationship breakdowns, unemployment and poverty (1). Preventing and mitigating the impact of these problems on parents and children is critical for improving population health for families now and in the future. However, no one sector or organization is in a position to address all the issues that these families may face. Hence, it is proposed that a “village approach” is needed when bringing up children.

The genesis for this perspective article comes from the *It takes a village*, an international conference held in Oslo, 2018. The conference brought together those with lived experience, researchers, practitioners and policy makers to discuss the needs of such families but arguably more importantly, optimal service responses. Given its audience, efforts were made, when putting together symposiums and accepting articles, to highlight ways the village might work together. Others also employ the term “village”, for example, the Austrian *How to raise the village to raise the child*, an initiative funded by the Ludwig Boltzmann Society and the Medical University of Innsbruck. The initiative aims to strengthen formal and informal support for children living with parental mental illness. Drawing on these initiatives, this article documents what is meant by the concept of a village approach. This article constitutes an attempt to “move toward” clarifying and discussing the concept of “the village” rather than provide a definitive set of guidelines or recommendations.

In this perspective, we first define what we mean by the “village” and then provide some discussion about what we mean by the term “families”. The need to move past traditional practice silos and how the village might work with families is then discussed using two, brief case studies.

## Defining the “Village”

The phrase “it takes a village to raise a child” originates from an African proverb and conveys the message that it takes many people (“the village”) to provide a safe, healthy environment for children, where children are given the security they need to develop and flourish, and to be able to realize their hopes and dreams. This requires an environment where children's voices are taken seriously (2) and where multiple people (the “villagers”) including parents, siblings, extended family members, neighbors, teachers, professionals, community members and policy makers, care for a child. All these ‘villagers' may provide direct care to the children and/or support the parent in looking after their children. However, the village, in many countries today, is dissipated and fragmented and individuals are increasingly isolated and are not eager to ask for, or provide help to, others. Family breakdown, economic pressures, long working hours and increased mobility have all contributed to families feeling less connected to extended family members and others around them (3).

In this perspective article, we propose a village that has the capacity to provide support and guidance to families living with adversity. Inherent in the concept of the village is the notion that caring for children is a shared responsibility amongst many. In this article we explore the notion of the village further, provide case studies of when it is occurring and provide principles of a village approach.

## Defining Family

Families mean different things for different people. Osher and Osher (4) suggested that family is “defined by its members, and each family defines itself” (p. 48). Likewise, Eassom et al. (5) provided a broad approach to the definition of family, which may not necessarily include one's biological family, but instead consists of those who share a common purpose, set of conventions and customs. Hence, there are different types of families, which may include the traditional nuclear (two parent) families, single parent families, adoptive families, same-sex parents, foster families, stepfamilies, and those in which children are raised by grandparents or other relatives.

One important role of families is to provide love, guidance, care, and support for its members. How they do this will differ, according to culture, family values, and the availability of educational, economic, and welfare resources. Through an interpretative framework, parents convey to their children the values, standards and rules about relationships and social structures. In turn, parents' beliefs and practices reflect the norms and expectations of their time and the culture in which they live. All of these factors impact the family environment and inform how family members show emotions, make decisions, resolve conflicts, interact with, and care for each other. When one family member is ill, facing addiction problems, or is otherwise under stress, other family members, including children, are inevitably impacted (1). In these instances, other family members may support the family member who is ill or under stress; alternatively (or in some instances, additionally), the family may itself be the source of trauma and ongoing stress and anxiety (6).

Multiple studies have shown that compared to other children, children growing up in such families may experience negative impacts on their own mental health and well-being, physical health and education (7). However, not all children whose families experience adversity will be negatively impacted, nor will all children be affected in the same way (1). Moreover, Gladstone et al. (8) argue that rather than being passive victims, many young people living in these families have their own agency, and in the face of great adversity, can be highly resilient and active contributors to family life.

Throughout recent history there have been different ways of describing families experiencing multiple adversities. In an address at a 1946 conference, Wofinden, a public health researcher, defined families who experience problems as “families with social defectiveness of such a degree that they require care, supervision and control for their own well-being and for the well-being of others” [(9), p. 127]. He continued by suggesting that “help from outside [the family] can hardly be of permanent value, except in proportion as it tends to develop the self helping faculties” (p. 130). In more recent times, public policy has mirrored similar sentiments. The 2011 Troubled Families Programme launched in England aimed to “turn around” the lives of the 120,000 most troubled families in England by 2015. In that policy, these “troubled families” were seen to “have” problems and “cause” problems to those around them (10). Such simplistic arguments condoned and extenuated the complex and interrelated relations between socioeconomic and psychosocial problems that many families experience, often over multiple generations. Such positioning also negates the responsibility of the “village” to support families. Helming et al. (11) consolidated such arguments when they write:

The concept of “multi-problem families” includes only the level of the family system (“families that have many problems”) and hides social deprivation [and] the deprivation of these families…. The term also neglects the… the obligation of the state to intervene to regulate equal opportunities (translated from the original, p. 74)

Tausendfreund et al. (12) advocated for the term “families in multi-problem situations” rather than “multi-problem families” so that the location of the “problem” is ascribed (semantically at least) to the family's environment rather than the family itself. Similarly, Goerge and Wiegand (13) employed the term “multisystem families”, though acknowledge that this only captures those problems that families seek assistance for, and that services are able to address. When responding to these families, Hayden and Jenkins (10) advocated for a two-prong government approach that involves: (i) providing immediate responses for supporting the whole family, and not only the individual adult and child “problems”, and (ii), targeting underlying driving forces behind family problems, especially pertaining to unemployment and insecure housing. Defining problems by the systems families engage with and the need to look at underlying forces, underscores the need for a village approach.

## Siloed Practice

Typically, organizational responses and policies for families living with adversity have been siloed, for example, supporting a parent presenting for cancer treatment without consideration of the needs of his or her children (14), or working with a client's mental illness without acknowledging his or her substance use problem (15, 16). Changing siloed practices is difficult, because they are grounded in professional development and education, laws and regulations, health policy and funding models (17).

Roberts (18) described silos as the “inability to share information and integrate system activity” (p. 677). Goerge and Wiegand (13) investigated families experiencing multiple adversities in the state of Illinois (USA) and found that 23% of families surveyed received services from two or more public services, including child welfare, mental health, substance abuse services and adult and juvenile incarcerations, mirroring findings from an audit of adult and child mental health services in Northern Ireland (19). Even though these families accounted for 86% of the funding for these services, there was little coordination or collaboration of care and little or no sharing of information between services. This siloed approach results in either an overlap of services or alternatively misses critical problems that a family may want and need to address. Siloed practice models are a problem that appears to be pervasive across countries, agencies and funding models (17).

Problems that may arise in families can correlate, for example, when parents who have a history of substance use also have a mental illness (20), or when one family member who has a mental illness has other family members who experience their own mental health issues (21). The reciprocal impact between children's and parents' health should not be underestimated (22) and will also reverberate in families. Exposure to one problem often leads to other problems, such as unemployment, inadequate housing, and in some cases violence and child neglect (1). Social complexity theory may help understand the problems families face; what might seem like chaotic behaviors are instead highly organized with rehearsed patterns. Complexity theory shifts attention from a “decontextualised and universalized essence to a concern with contextualized and contingent, complex wholes” [(23), p. 119]. This necessitates looking past presenting behaviors (e.g., the reaction of children to a parent's symptomology) and instead, appreciating the ways in which interactions with others, material resources and services contribute to family experiences. Rather than see families as dysfunctional or beyond hope, we need to recognize that they may be striving for meaning and balance and doing the best that they can, in their given circumstances (24).

The complexities of these adversities further underscore the need for coordinated responses across health, housing, employment services, education, policing and other agencies and community groups, from the perinatal period through to adulthood. Different services will be needed at different times, especially for key developmental milestones, such as the birth of a new child or the death of a grandparent (25). Moreover, the impact of these adversities can be intergenerational, as the impact of the adversity is passed on through parenting practices, violence, substance misuse and mental health issues (26).

## Social Connectedness

Even though parents may be a child's primary caregivers, a family does not exist in a vacuum. Social connectedness has been defined as those subjective psychological bonds that people experience in relation to others including, for example, a sense of belonging and feeling cared for (27). It also includes objective measures such as the frequency of social participation and marital status (27). There is much evidence that strong, positive connections are linked to positive mental health and well-being, especially in times of stress or trauma (27). Social connectedness is one way of describing the members of the village and the need for families to have multiple supports. Given that responding to, and overcoming adversity, occurs in a social context that extends beyond individual and family levels, social connections for families living in adversity includes but extends past members of the immediate family.

There are, however, many families who are not included within their communities. Likewise, families with complex health and social needs may be excluded from services, for many reasons, leading to poor health outcomes and multiple morbidities and in some cases early mortality (28). Families may be excluded because of the stigma associated with adversity (such as mental illness or poverty) and an inappropriate representation in the media (29) or because they are not recognized by a government's criteria of “troubled”, and are missing from public policy (10). Rather than being “hard to reach”, some families may not have the ability to access services (because of transport or time), may have had negative experiences with similar services previously and/or find them intimidating or unhelpful. Some may not be aware of services that could assist them and may need professionals to serve as conduits to other services (30). The village concept implies a need to identify the magnitude of exclusion (that is, who is being excluded and from what), specify why they are excluded and, on that basis, promote access to essential services for individuals and their families and challenge societal attitudes and media misrepresentations. Families need different forms of connections, formal and informal, from the individual level to the policy and government level, to address the upstream causes of exclusion and disadvantage, including adverse childhood experiences and poverty.

## Who is in the Village?

**Figure 1:**
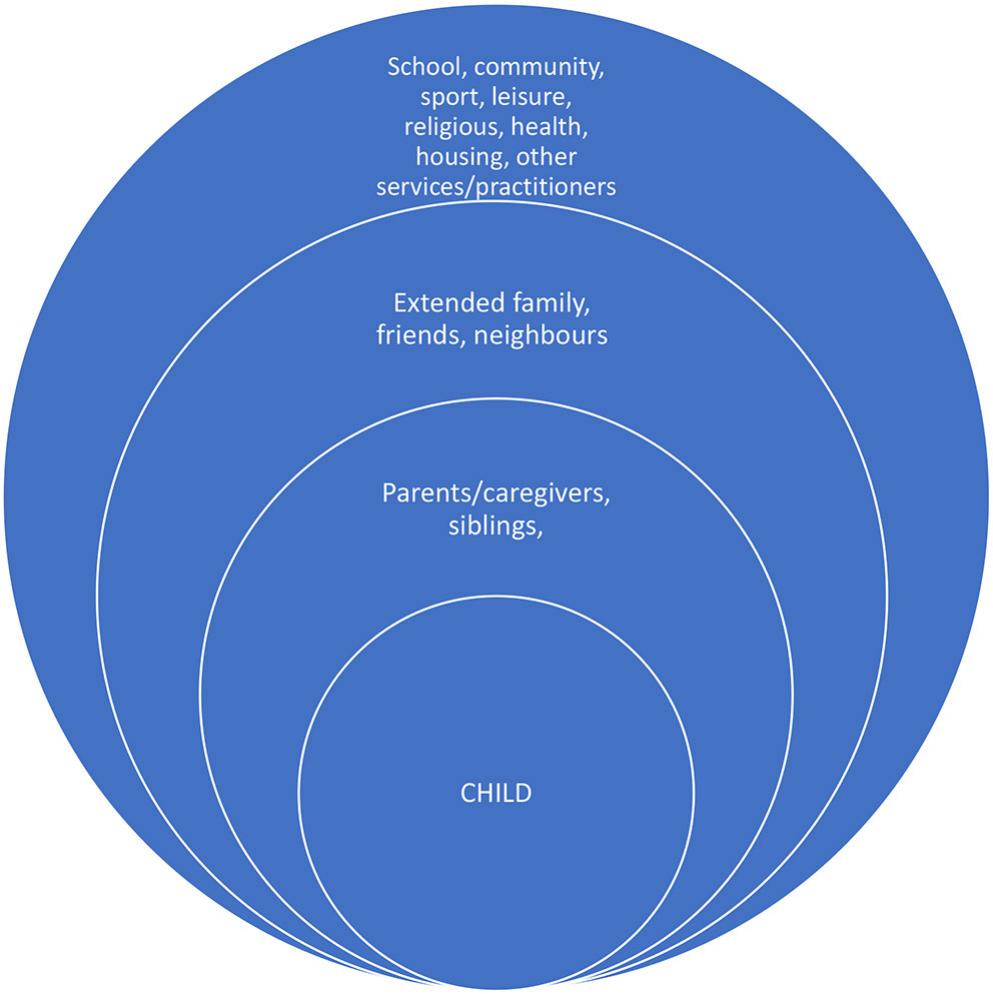
Possible connections for families living in adversity.

Bronfenbrenner's ecological theory (31) highlights the various factors that impact on children's learning and development. We have extrapolated from that model to highlight the connections that families might have, in each sphere, as one possible indication of who might be the “villagers” (see Figure 1). This figure demonstrates how different social connections impact children's outcomes, across varying proximity levels (though this may also vary for different families). Culture, socioeconomic status and language provide further context to this figure. It is the richness (quality and quantity) of these connections that can have a significant influence on the quality of the child rearing that a parent provides and the types of connections that children might make (32). Synergy is an important aspect of this model, which implies that families, schools, community groups and agencies working together can achieve more than either could alone (33).

Supportive connections with village members are valuable for both children and parents. In her seminal longitudinal study of high-risk children, Werner (34) found that children from high risk backgrounds, who formed bonds with caring and trusting other adults, turned out to be more resilient, than those who did not form such connections. Connections also help parents; Garbarino and Sherman (35) found that parents who have access to social networks and supports when looking after children report less parenting stress, than other parents. Likewise, communities with strong formal and informal networks are associated with lower rates of child maltreatment, compared to communities characterized by social disorganization and low levels of social cohesion (36).

Though the importance of social connections might be self-evident, Kesselring (32) argued that in western societies there is a trend toward parenting as a private concern, and when any presenting problems (experienced by the child or family) are referred to professionals rather than shared amongst the family's social networks. In this approach, the village shrinks considerably, especially when professional services are limited or are not accessed by the family (for whatever reason). However, in many societies, nonparental caretaking is either the norm or occurs frequently. Donner (37) found that in Polynesian society both parents and nonparents were involved in the upbringing of other people's children. Polynesian adults viewed the western ideal of sole parental responsibility as a “lack [of] compassion” for other people's children (p. 703). Likewise, Otto (38) found that Cameroonian Nso mothers discouraged maternal exclusivity, believing that multiple caregivers are optimal, with one mother stating, “Just one person cannot take care of a child throughout” (p. 95).

There are, however, times when parents in western cultures draw on different members of the village. In the UK, Edwards and Gillies (39) found that although many parents receive less informal support than in the past (because of divorce, or because extended families are geographically dispersed), parents still identified relatives and friends as the main source of emotional support and advice about their children's behavior. In the USA, Burchinal et al. (40) found that in communities where neighbors trust each other, parents are more likely to utilize informal childcare from their neighbors, rather than relying exclusively on their relatives to look after their children, when working or ill. Both neighbors and parents can be involved in caring for children when they have “shared expectations and mutual engagement by adults in the active support and social control of children” [(41), p. 635]. Professionals, such as teachers and youth workers, play a role in these neighborhoods by organizing neighborhood activities and events and by “caring” for children (42). Governments in many western countries focus on parenting in public provision and policy, and provide some families with government financial support and information and hands-on support through different initiatives and parenting programs (43). There are also different parenting blogs and other online sites that parents might access, to meet other parents and/or obtain emotional support and advice. In sum, the different connections that a family might make (Figure 1) help us understand the different forms of support that may be provided, and those that may be missing.

## Potential Principles of the Village Approach

The connections within the village approach are important, but how these connections might confer protection or buffer the impact of adversity is not always clear. Articulating principles for a village approach serves as the first step in operationalising the village approach. Based on our collective experiences as researchers and clinicians, these principles have been outlined in Table 1, along with practice and/or policy implications. These principles might be used to develop new initiatives and evaluate existing ones, an important future direction in the field.

**Table 1:** Potential principles of the village approach.

**Village principle**	**Practice and policy implication**
Interdisciplinary	Practitioners from various professional disciplines, including but not limited to physical health, psychology, social work, and education, are provided with the training and time to work collaboratively
Interagency	Coordinated interagency support is provided to families depending on need, including but not limited to housing, employment, child care and education
Strength based	Family, parenting and children's strengths and resources are identified, recorded and celebrated.
Prevention focused	Support aims to prevent immediate and long-term problems.
Developmental, lifespan approach	Different support is provided to parents/caregivers and children at different times, depending on key developmental milestones.
Promoting parents' agency and empowerment	The views and perspectives of parents is actively sought when defining problems and solutions. Parents are partners in the planning and delivery of services.
Giving children a voice	Children of all ages are encouraged to present their perspectives on the issues and potential solutions to existing and future family issues
Culturally sensitive	Individual, familial and communal cultures are acknowledged and considered when addressing problems and solutions.
Feedback and evaluation	Feedback and evaluation processes are built into Village-focused policies and practices

Applying these principles in practice is the next step to which might challenge the social factures that inhibit the notion of the village that may intentionally or unintentionally exclude families. In this final section we provide two case studies which demonstrate the ways in which “the village” might be enacted.

### Harlem Children's Zone

Aiming to improve the educational and developmental outcomes for children in one of America's most impoverished communities, the Harlem Children's Zone (HCZ) is a non-profit organization for children and families that includes community building, the promotion of parent networks and neighborhood safety, and child-oriented education and health programs (44). By promoting a sense of community, HCZ addresses a constellation of factors that might negatively impact families. Individual programs may be delivered, for example, that focus on housing, but these incorporate a mandate to foster community connections and support healthy physical and social environments. HCZ services are structured into a “pipeline” of continuous support from a child's birth through to college graduation. Services include parenting supports, which provide a safe space for parents to connect with others and provide information on parenting best practices and pathways to coordinate and navigate services. Evaluations indicate that HCZ significantly increased academic achievement for children living in adversity (45), and has positive impacts on children's weight and physical health (46).

The HCZ incorporates many of the principles covered by the village approach (Table 1) by providing an interdisciplinary, interagency approach and before that is prevention and youth focused. The “pipeline” of supports is clearly developmentally orientated and its focus on parenting support promotes parents' agency and autonomy. Nonetheless, there have been calls for further evaluation to demonstrate the efficacy of this approach, especially in regards to impacts on children's well-being, in addition to their academic outcomes (44).

### Strategies With Kids–Information for Parents

Developed in New Zealand, SKIP is a government funding program that aims to increase the opportunities for communities to promote positive parenting, for families living in adversity (47). SKIP employs an open tender process in which the government invites local organizations and groups to submit proposals that aim to support families in a holistic, culturally sound manner. For example, one initiative brings parents together to share successful strategies for positively managing challenging behavior in their preschool children, while another identifies community and agency partnerships for addressing community violence. Its approach affirms the role of parent and provides the pathways for normalizing help seeking in communities, in culturally appropriate ways. The initiative's locally driven, strength-based approach aligns with the village principles (Table 1) as does its focus on promoting parents' agency and empowerment. However, children's voices appear to be lacking as is any form of rigorous evaluation and monitoring.

The two case studies illustrate community led approaches that aim to promote the development of a village approach that benefits children and their families. Both demonstrate the large number and range of initiatives offered, which draw on existing capacity and address the specific needs of the local community. The most common activities appear to be the active involvement of parents in the planning and development of programs, active community engagement, and promoting safe, family friendly environments. The potential to use community settings, such as schools, to upscale interventions is also evident, allowing local communities to drive programs adapted to their context within existing resources.

The Austrian project mentioned at the start of this perspective, titled *How to raise a village to raise a child*, has a program theory model that the authors argued promotes the capacity of the village to care for children and families; this model outlines resource inputs, systems and individual context considerations and triggers for behavior changes (48), with a particular focus on translation and implementation (49). A subsequent article further emphasized the importance of regional context specific solutions and engagement with local and experienced stakeholders to ensure service models are implementation ready (50). As the authors themselves conceded, their work to date has not yet demonstrated the effectiveness of this approach, especially in terms of child outcomes (50). A major issue in the field appears to be that many of these broad community projects have not been rigorously evaluated, especially in regard to how children may benefit. Some of the reasons for this, at least partially, may be that the principles of a village approach are rarely articulated, hence the need for this article. Additionally, as Nicholson (51) argued, the complexity involved in an ecological model of family functioning makes gold standard evaluations (typically employing a randomized controlled trial) difficult to conduct; we would suggest that a village approach makes conducting an evaluation even more challenging but one that researchers are currently addressing [see for example, (48)].

Many of those who organized and participated in the 2018 *It takes a village* conference, were involved in the writing of an editorial which outlined various recommendations for systems and workforce change, (52) and which generated much traditional and social media interest. The recommendations article, the two case studies shown, and the recent Austrian project, indicate that there is interest in the concept of the village. However, further research is required to demonstrate how a village approach might be enacted in different settings and with different families, and in particular, evaluating its long term impact on families.

## Conclusion

This article provided one perspective of a “village” approach when supporting families who experience various challenges. We describe a village approach, which ranged from immediate child and family level responses through to government lead initiatives that services and governments might need to consider when developing practice guidelines and public health policy. The connections and principles identified in this perspective might serve as the framework from which new initiatives could be developed and existing programs evaluated. These connections and principles are even more pertinent given the struggles experienced by families and communities throughout the COVID-19 pandemic (53).

## Author Contributions

All authors were responsible for the conceptualization of the article and contributed to its writing.

## Conflict of Interest

The authors declare that the research was conducted in the absence of any commercial or financial relationships that could be construed as a potential conflict of interest.

## Publisher's Note

All claims expressed in this article are solely those of the authors and do not necessarily represent those of their affiliated organizations, or those of the publisher, the editors and the reviewers. Any product that may be evaluated in this article, or claim that may be made by its manufacturer, is not guaranteed or endorsed by the publisher.
